# Complete duplex ureter with ureterocele and multiple large stones in the lower ureter: a case report

**DOI:** 10.1093/jscr/rjaf850

**Published:** 2025-10-24

**Authors:** Zhicheng Luo, Shangjun Wu, Jie Deng, Huiling Jiang, Xiqi Peng

**Affiliations:** Department of Urology, South China Hospital, Medical School, Shenzhen University, No. 1 Fuxin Road, Longgang District, Shenzhen 518116, China; Department of Urology, South China Hospital, Medical School, Shenzhen University, No. 1 Fuxin Road, Longgang District, Shenzhen 518116, China; Department of Urology, South China Hospital, Medical School, Shenzhen University, No. 1 Fuxin Road, Longgang District, Shenzhen 518116, China; Department of Urology, South China Hospital, Medical School, Shenzhen University, No. 1 Fuxin Road, Longgang District, Shenzhen 518116, China; Department of Urology, South China Hospital, Medical School, Shenzhen University, No. 1 Fuxin Road, Longgang District, Shenzhen 518116, China

**Keywords:** ureteral duplication, ureterocele, stone, laser lithotripsy, ureteral access sheath

## Abstract

Complete duplex ureter with ureterocele and multiple large ureteral stones is a rare congenital anomaly that can be easily misdiagnosed as bladder calculi. We report a 48-year-old woman who presented with urinary frequency and was preoperatively diagnosed with bladder stones. However, intraoperative ureteroscopy revealed a complete duplex ureter on the right side with a ureterocele in the lower segment harboring multiple large stones. A Y-shaped ureteral access sheath (UAS) was used as a working channel, enabling effective holmium laser lithotripsy and fragment evacuation. This case highlights the importance of considering ureteral anomalies in atypical stone presentations and suggests that modified UAS devices may facilitate safe and efficient treatment in complex anatomical settings.

## Introduction

Duplex collecting system is a common congenital urogenital anomaly caused by the incomplete fusion of upper and lower pole of kidney, with an incidence of 0.5%–3.0% [1]. Depending on the degree of ureter fusion, duplication can present as single, bifid (partial duplication), or complete double separate ureters inserting to the bladder, with the complete form being less common [2]. Although ureteral duplication is typically asymptomatic, it may lead to complications such as infection, reflux, or obstruction when accompanied with ureterocele, calculi, or other congenital anomalies. The diagnosis of ureteral duplication is often incidental during routine diagnostic evaluation, as its symptoms often mimic other urinary disorders and thus be overlooked.

Our case describes a complete duplex ureter with ureterocele in the lower ureter and multiple large stones without hydronephrosis. Preoperative examination indicated the preliminary diagnosis as bladder stone. The final definitive diagnosis was established intraoperatively and the calculi were resolved through a tailored endoscopic approach.

## Case report

**Figure 1:**
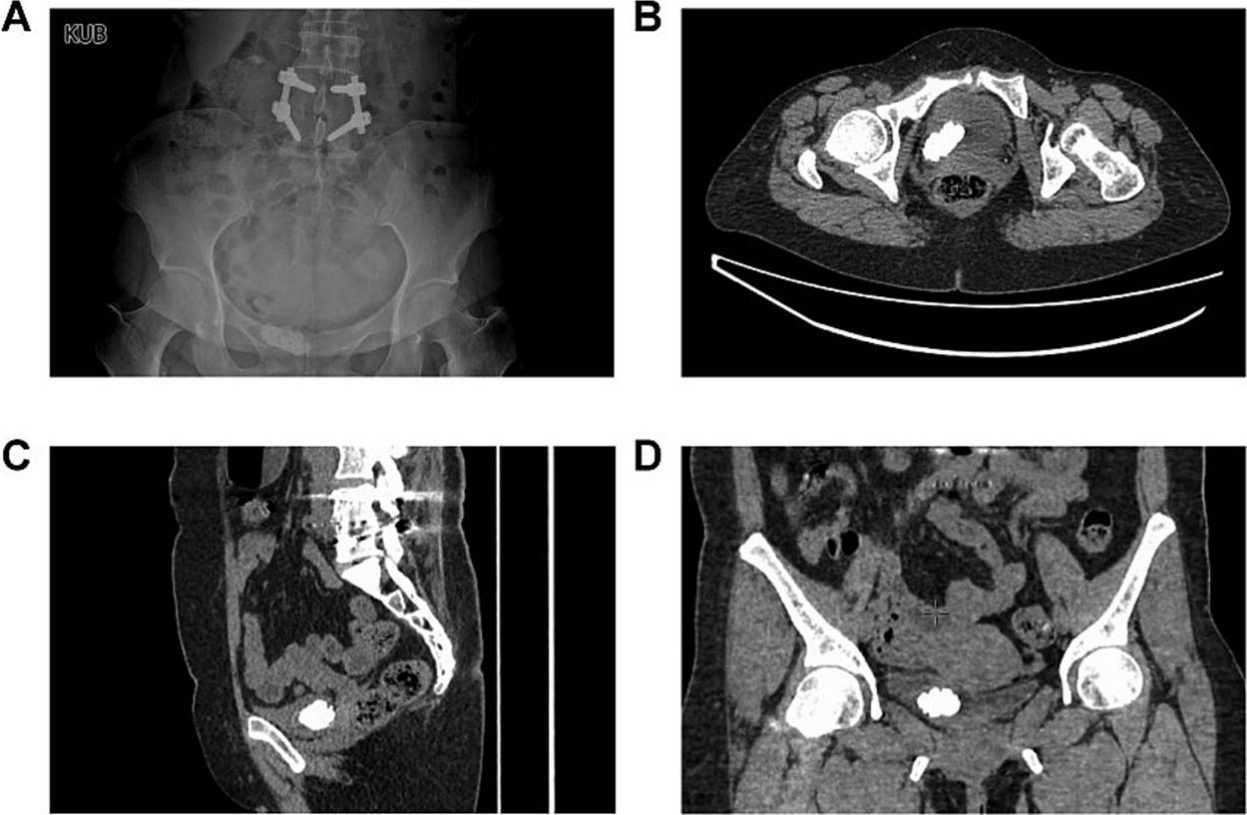
The preoperative imaging examinations of the patient. (A) The plain film of kidney–ureter–bladder. (B–D) Computed tomography.

A 48-year-old female was admitted with a diagnosis of “multiple bladder stones” based on color doppler ultrasound (CDU) examination. The patient complained of frequent urination without back pain or fever. Ultrasound revealed no hydronephrosis in both sides. The patient had a history of lumbar surgery and cesarean section. Upon admission, no kidney percussion pain was noted. Blood routine tests, renal, and coagulation function tests were normal. Urinalysis showed leukocytosis, and urine culture was negative. Kidneys, ureters, and bladder (KUB) imaging reported a stone in the pelvic area (Fig. 1A). Computed tomography scan (CT) reported a large 4 cm “bladder stone” and multiple stones in the right kidney (Fig. 1B–D).

On 9 July 2024, transurethral holmium laser lithotripsy was performed. During ureteroscopy no bladder stones were observed. Instead, we observed two separate ureteral orifices (a and b) on the right side and one ureteral orifice on the left side.

**Figure 2:**
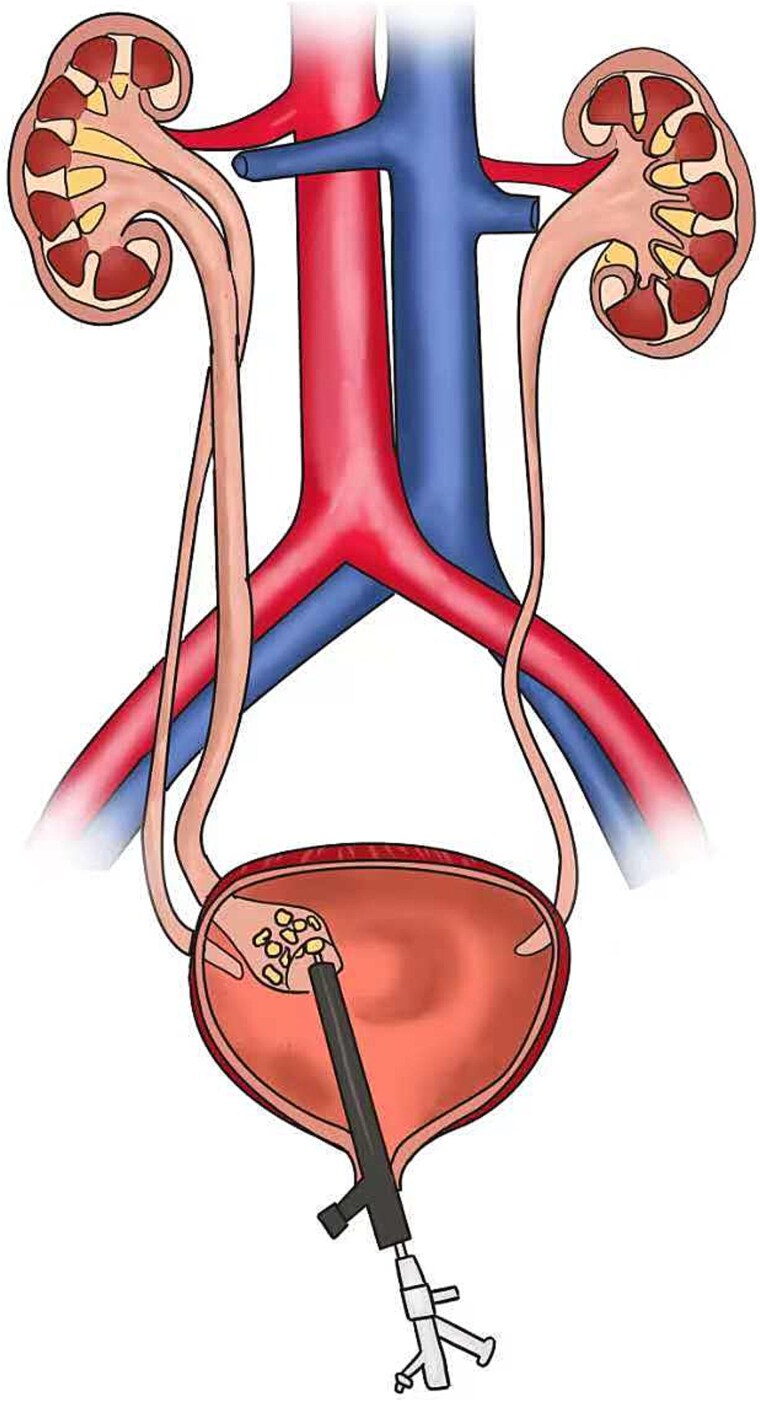
Schematic diagram of the surgery process.

**Figure 3:**
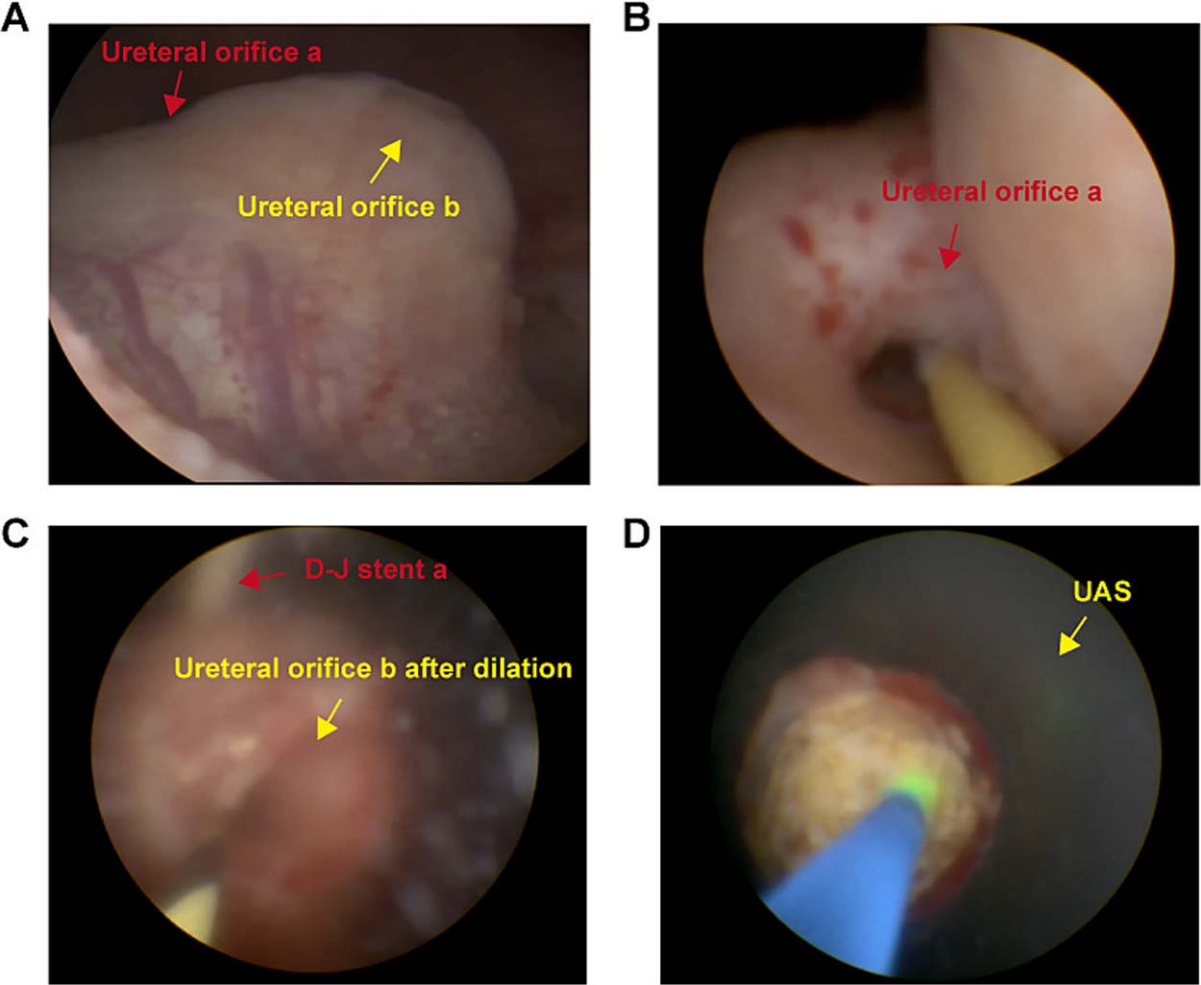
Intraoperative images revealing duplex ureter with ureterocele harboring multiple large stones. Multiple stones were discovered at the opening of ureter b and the surgery was assisted by UAS.

Ureteroscopy via the right ureteral orifice a found no stones and a DJ stent was placed after the examination. Ureteroscopy via the right ureteral orifice b found cystic dilation in the lower segment of the ureter, with a large number of stones within the lower segment of the ureter (Fig. 2). No stone was observed in the upper segment of the right ureter. The endoscope was exited with the guide wire retained, and the Y-type ureteral access sheaths (UAS) (ClearPetra Nephrostomy Access Sheath Set,16/18Fr × 15 cm) were inserted along the guide wire to expand the ureteral orifice. The 16Fr Sheath was retained as the working channel (Fig. 3).

**Figure 4:**
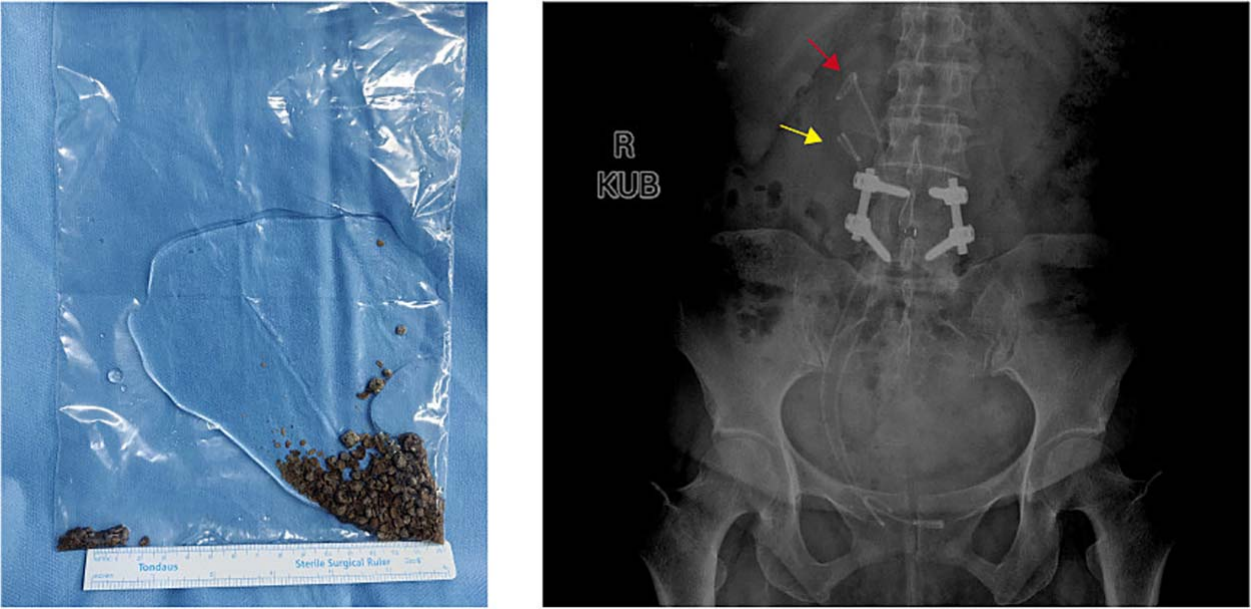
Postoperative stone display and KUB imaging examination.

Holmium laser was used for lithotripsy, and the fragments were removed by negative pressure suction. Another DJ stent was placed via orifice b. The total operation time was 153 min. Postoperative KUB confirmed complete lithotripsy and correct stent in the duplex ureters (Fig. 4). The patient was discharged 3 days after surgery. All DJ stents were removed 1 month later without complications. The patient declined further examination and treatment for kidney stones and ureteral anomalies. No discomfort was reported during 6-month follow-up.

## Discussion

Ureteral duplication, a common congenital urinary abnormality, is usually accompanied with complications such as urolithiasis, ureteroceles, infections, and vesicoureteral reflux. The unique anatomy of duplicated kidney and ureteral malformations, along with ureteral stenosis and poor urine drainage, is prone to reflux and infections, thereby increasing the risk of stone formation [3, 4].

CT and MR urography assist the diagnosis of ureteral duplication, enabling the differentiation between complete and incomplete duplication patterns through visualization of the entire ureteral trajectory. Intravenous urography (IVU) may demonstrate duplicated ureters and associated ureteroceles, particularly through the pathognomonic “cobra head” sign in patients with preserved renal function. However, when ureteral duplication combined with other complications such as urolithiasis, clinicians often routinely use color doppler ultrasound or routine CT for evaluation, making collecting system anomalies easy to overlook [5, 6].

Urolithiasis burden in the cases of complete duplex ureter with ureterocele can be substantial. The treatment is more complicated than normal lower ureteral stones due to anatomical variation and high urolithiasis burden. Minimizing postoperative complications during lithotripsy is essential. Currently, there are no common standards for treatment of these cases. Routine rigid ureteroscope surgery is adequate in cases with small stones [7]. For those more complicated cases, one simple and effective way is to directly remove the stones through the incision of ureteroceles [8]. Some surgeons suggest the laparoscopic ectopic ureterectomy and replantation of the ureter to address the problems of calculi and ureterocele deformity at the same time [9, 10].

Rigid ureteroscope remains the standard for the treatment of lower ureter stones. During lithotripsy, continuous saline infusion is required to ensure visualization and reduce thermal damage from the holmium laser. However, sustained high irrigation pressure may cause bloodstream translocation of bacteria and endotoxins，volume overload, and potential renal damage [11]. Therefore, in high stone burden cases, it is important to increase the efficiency of stone removal with controlled intrarenal pressure (IRP) via proper methods or tools [12], for the purpose of reducing post-infection complications, shortening operative time and increasing stone-free rate.

This case introduces a complete duplex ureter with ureterocele and multiple large stones in the lower ureter. While we unexpectedly observed ureterocele with multiple stones instead of our preoperative diagnosis of bladder stones during operation, leveraging our experience, we applied a Y-shaped PCNL sheaths as UAS to provide a better working channel. The working sheath helped maintaining IRP and effectively flushing out stone fragments to reduce the risk of urethral injury and the length of operation.

Complete duplex ureter with ureterocele and large stones is rare and easily misdiagnosed. In the case of large urinary tract stones near the ureteral orifice without hydronephrosis and LUTS, complete duplex ureter with ureterocele should be considered. In such cases, the use of a UAS such as Y-shaped PCNL sheaths may offer a novel approach. Clarifying the indications and sheath sizing for UAS may contribute to more effective and standardized treatment strategies in patients with large stone burden in complex duplex systems.
